# MALT1 inhibitors prevent the development of DSS-induced experimental colitis in mice via inhibiting NF-κB and NLRP3 inflammasome activation

**DOI:** 10.18632/oncotarget.8867

**Published:** 2016-04-20

**Authors:** Wen Liu, Wenjie Guo, Nan Hang, Yuanyuan Yang, Xuefeng Wu, Yan Shen, Jingsong Cao, Yang Sun, Qiang Xu

**Affiliations:** ^1^ State Key Laboratory of Pharmaceutical Biotechnology, School of Life Sciences, Nanjing University, Nanjing 210093, China; ^2^ Eternity Bioscience Inc, Cranbury, NJ 08512, USA

**Keywords:** colitis, MALT1, IL-1β, NF-κB, NLRP3 inflammasome

## Abstract

Mucosa-associated-lymphoid-tissue lymphoma-translocation gene 1 (MALT1), a paracaspase and essential regulator for nuclear factor kB (NF-κB) activation, plays an important role in innate and adaptive immunity. Suppression of MALT1 protease activity with small molecule inhibitors showed promising efficacies in subtypes of B cell lymphoma and improvement in experimental autoimmune encephalomyelitis model. However, whether MALT1 inhibitors could ameliorate colitis remains unclear. In the present study, we examined the pharmacological effect of two specific MALT1 inhibitors MI-2 and mepazine on the dextran sulfate sodium (DSS)-induced experimental colitis in mice, followed by mechanistic analysis on NF-κB and NLRP3 inflammasome activation. Treatment with MI-2 and mepazine dose-dependently attenuated symptoms of colitis in mice, evidenced by reduction in the elevated disease activity index, the shortening of colon length as well as the histopathologic improvement. Moreover, protein and mRNA levels of DSS-induced proinflammatory cytokines in colon, including TNF, IL-1β, IL-6, IL-18, IL-17A and IFN-γ, were markedly suppressed by MALT1 inhibitors. The underlying mechanisms for the protective effect of MALT1 inhibitors in DSS-induced colitis may be attributed to its inhibition on NF-κB and NLRP3 inflammasome activation in macrophages. The *in vitro* study showed that MALT1 inhibitors decreased production of IL-1β/IL-18 in phorbol myristate acetate-differentiated THP-1 cells and bone marrow derived macrophage via suppressing the activation of NF-κB and NLRP3 inflammasome. Taken together, our results demonstrated that inhibition of the protease activity of MALT1 might be a viable strategy to treat inflammatory bowel disease and the NLRP3 inflammasome and NF-κB activation are critical components in MALT1 signaling cascades in this disease model.

## INTRODUCTION

Ulcerative colitis is a chronic inflammatory disorder in the gastrointestinal tract. It has a high prevalence worldwide and is a well-established risk factor of colorectal cancer [1, 2]. Although the etiology of the disease is unknown, it has been suggested that the activation of the mucosal immune system in response to bacterial antigens with consecutive pathologic cytokine production plays a key pathogenic role [3] and increased expression of proinflammatory genes were frequently characterized in inflammatory bowel disease and experimental intestinal inflammation [4–6].

The synthesis and secretion of proinflammatory cytokines is governed by germline-encoded receptors such as the toll-like receptor (TLR) and nucleotide-binding domain leucinerich repeat containing (NLR) protein family [7]. As we known, NF-κB signaling-mediated macrophage activation is the main sources of inflammatory factors, which are related to IBD-associated inflammation [8–10]. While at the same time, many studies also demonstrated that NLRP3 inflammasome-mediated IL-1βand IL-18 release were involved in experimental colitis [11–14], suggesting that both NF-κB and NLRP3 axis may serve as potential targets for the development of novel therapeutics for patients with inflammatory bowel diseases.

Mucosa-associated-lymphoid-tissue lymphoma-translocation gene 1 (MALT1), as a scaffold protein, together with B-cell chronic lymphocytic leukemia/lymphoma 10 (BCL10) and caspase-recruitment domain containing membrane-associated guanylate kinase protein 1 (CARMA1) formed preformed complex, which mediates the recruitment of TRAF6 and other downstream molecules to activate the IκB kinase (IKK) complex, leading to the release and activation of NF-κB [15, 16]. At the same time, MALT1 could function as a protease activity upon TCR/CD28 co-stimulation to inactivate negative regulators of NF-κB signaling such as the A20 (also known as TNFAIP3), CYLD (cylindromatosis), RNase Regnase-1 as well as RelB and HOIL1 [17–21]. Generally, as a scaffold MALT1 assembles downstream signaling proteins for NF-κB activation, while its proteolytic activity further enhances NF-κB activation by cleaving NF-κB inhibitory proteins.

A tumor-promoting role of MALT1 has first been found in MALT lymphoma and activated B cell-like diffuse large B cell lymphoma (ABC-DLBCL) [22, 23]. And the following research has also proved that MALT1 is an intrinsic regulator of regulatory T cells [24, 25]. Based on this, the MALT1 inhibitor was developed and aimed for cancer therapy. The phenothiazine derivative mepazine was shown to act as a potent noncompetitive by binding to an allosteric pocket on MALT1 [26] and can inhibit the cleavage activity of recombinant and cellular MALT1. Consequently, the compounds significantly inhibited growth of ABC-DLBCL *in vitro* and *in vivo* [27]. Moreover, compound MI-2 has been identified as a selective MALT1 inhibitor, engaging and irreversibly binding the active site of MALT1 [28]. Conor *et al.* found out that mepazine can significantly protect mice in a mouse model of multiple sclerosis [29], indicating a possible use also for the treatment of severe autoimmune diseases.

Therefore, we herein assessed the potential of MATL1 inhibitors on the development and progression of DSS-induced colitis. We demonstrate the MATL1 inhibitors ameliorate clinical symptoms and histopathologic features of DSS-induced colitis via inhibiting NF-κB and NLRP3 inflammasome activation in macrophage *in vivo* and *in vitro*.

## RESULTS

### MALT1 inhibitors attenuated DSS-induced experimental colitis

**Figure 1:**
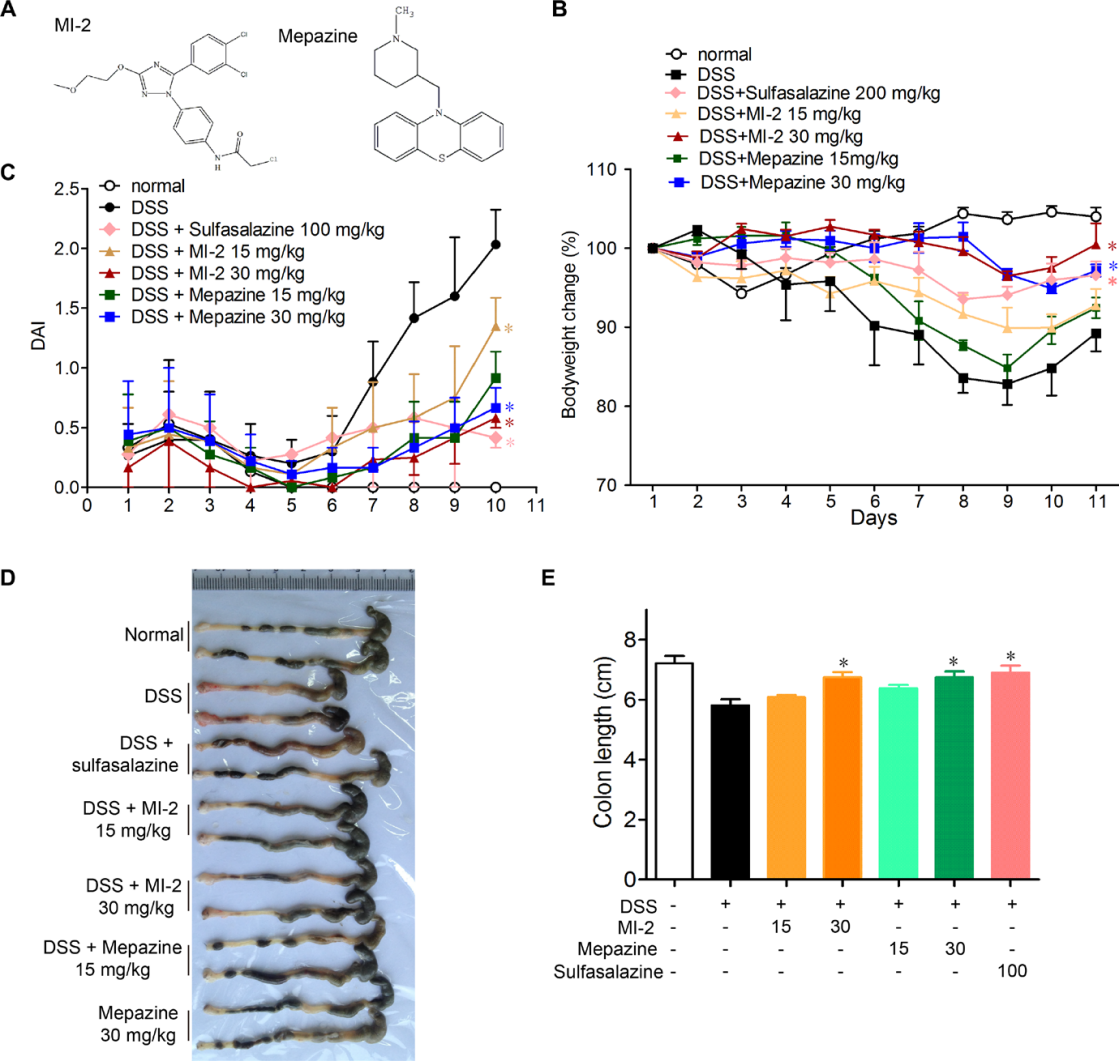
MALT1 inhibitors treatment ameliorated DSS-induced experimental colitis in mice (**A**) Chemical structure of MALT1 inhibitors MI-2 and Mepazine. Mice were given 2.5% DSS in drinking water for 7 days, then mice were provided with water for another 3 days before sacrificed. The mice in each group were treated with MI-2 or Mepazine (i.p., 15 and 30 mg/kg) or sulfasalazine (i.g., 200 mg/kg) for day 0–10. (**B**) Bodyweight change and disease activity index (DAI) (**C**) were calculated (*n* = 6 per group). (**D**, **E**) Macroscopic appearances and the length of colons from each group of mice were measured. Data are presented as means ± SEM. *P < 0.05, **P < 0.01 vs. DSS-treated alone group at the same day. ^#^*P* < 0.01 vs. normal group.

**Figure 2:**
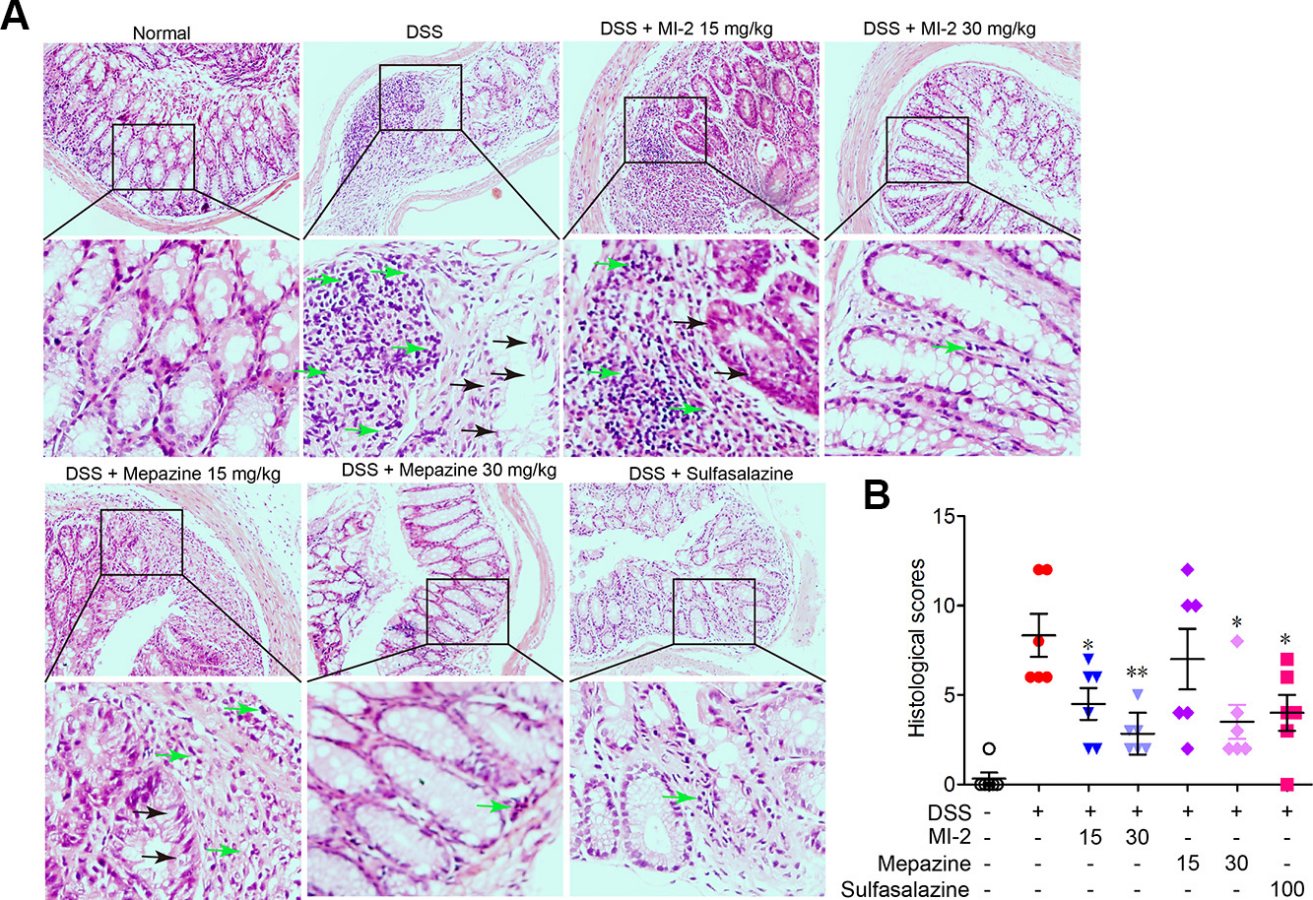
MALT1 inhibitors treatment prevented DSS-induced colon damage in mice (**A**) Serial sections of colon tissues were stained with H&E. Green arrow indicated mononuclear cell infiltration and black arrow indicated globet cell damage. (**B**) Histopathological scores of each group were determined. Data are presented as means ± SEM. *P < 0.05, **P < 0.01 vs. DSS-treated alone group at the same day. ^#^*P* < 0.01 vs. normal group.

To examine whether MALT1 inhibitors could be used for treating colitis, we first examined the effects of known MALT1 inhibitors mapzine and MI-2 in an animal model induced by DSS drinking. It is well known that DSS induces a severe illness in mice characterized by a dramatic loss of body weight, significant appearance of diarrhea/loose feces and visible fecal blood as evaluated by DAI (disease activity index). Compared with vehicle-treated group, MALT1 inhibitors mapzine and MI-2 (Figure 1A) at both 15 and 30 mg/kg significantly attenuated the loss of bodyweight and elevation of DAI during the disease progression (Figure 1B, 1C). As a drug used in clinical for colitis therapy, Sulfasalazine at 100 mg/kg also improved the DAI (Figure 1C). DSS typically causes colonic shortening, and such change was also improved by treatments with mapzine or MI-2 at 15 or 30 mg/kg (Figure 1D, 1E). Histological analysis showed distortion of crypts, loss of goblet cells, infiltration of mononuclear cells, and severe mucosal damage in the colon specimens of colitis mice (Figure 2A). Such histopathological abnormities were largely restored in mice treated with 15 or 30 mg/kg of mapzine and MI-2 (Figure 2B).

### MALT1 inhibitors regulated the cytokine profiles in colons of mice with DSS-induced colitis

**Figure 3:**
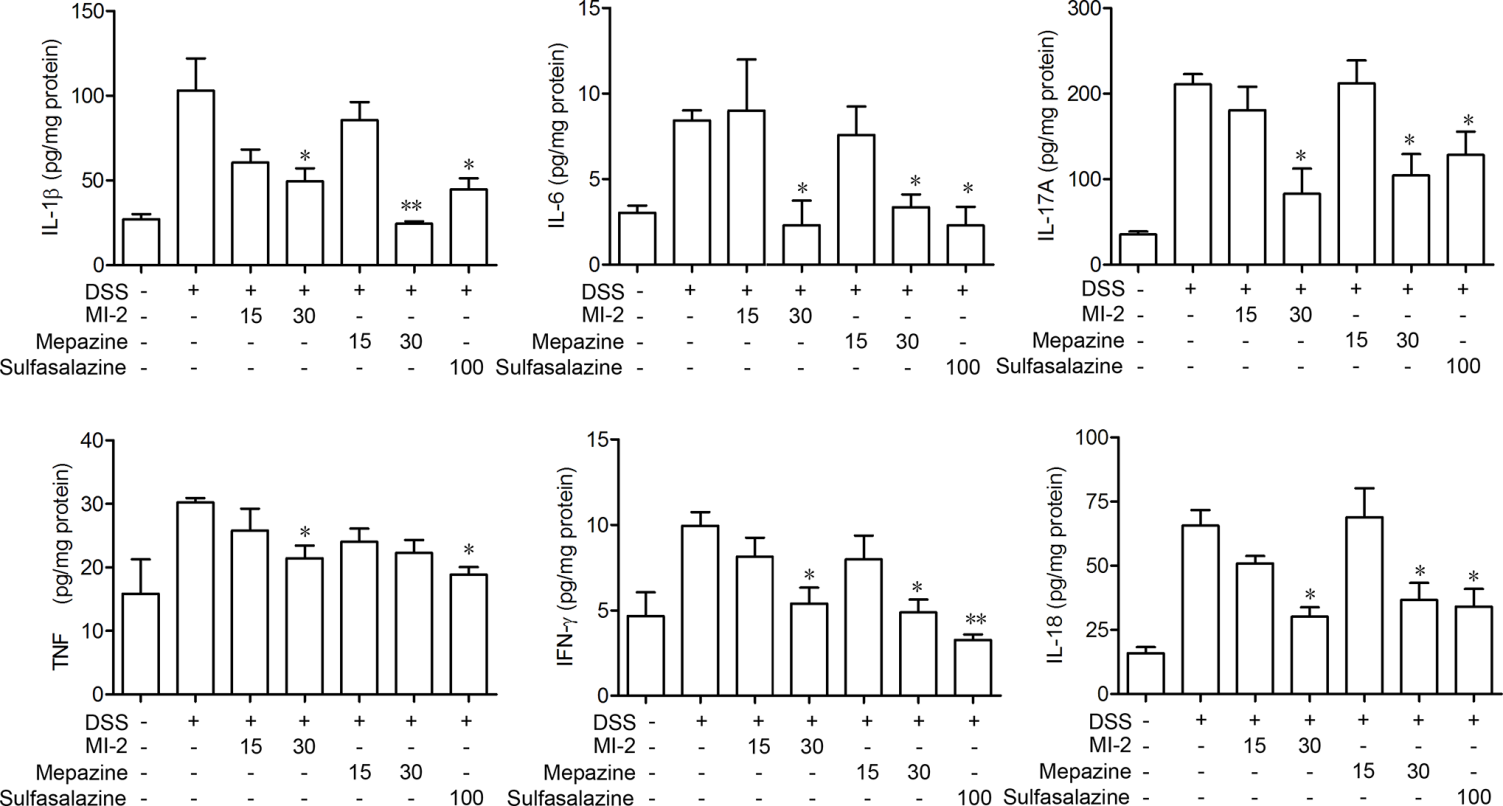
MALT1 inhibitors suppressed proinflammatory cytokine production in colon tissues from DSS-colitis mice Protein levels of cytokines including IFN-γ, TNF-α, IL-1β, and IL-6 in colonic homogenate were determined by ELISA. Data are presented as means ± SEM (*n* = 6). *P < 0.05, **P < 0.01 vs. DSS-treated alone group.

To examine the effects of MALT1 inhibitors on the cytokine expression in acute DSS colitis model, we measured the levels of IL-1β, IL-6, TNF, IFN-γ, IL-17A and IL-18 in colons of mice following induction of colitis and treatments with MALT1 inhibitors. As shown in Figure 3, ELISA analysis for the cytokine levels in colonic homogenated protein from each group showed that IL-1β, IL-6, TNF, IFN-γ, IL-17A and IL-18 were remarkably increased after DSS challenge. MI-2 and mapzine at 30 mg/kg can significantly inhibit the elevation of all cytokines in colon after DSS challenge.

### MALT1 inhibitors reduced DSS-induced activation of NF-κB signaling

**Figure 4:**
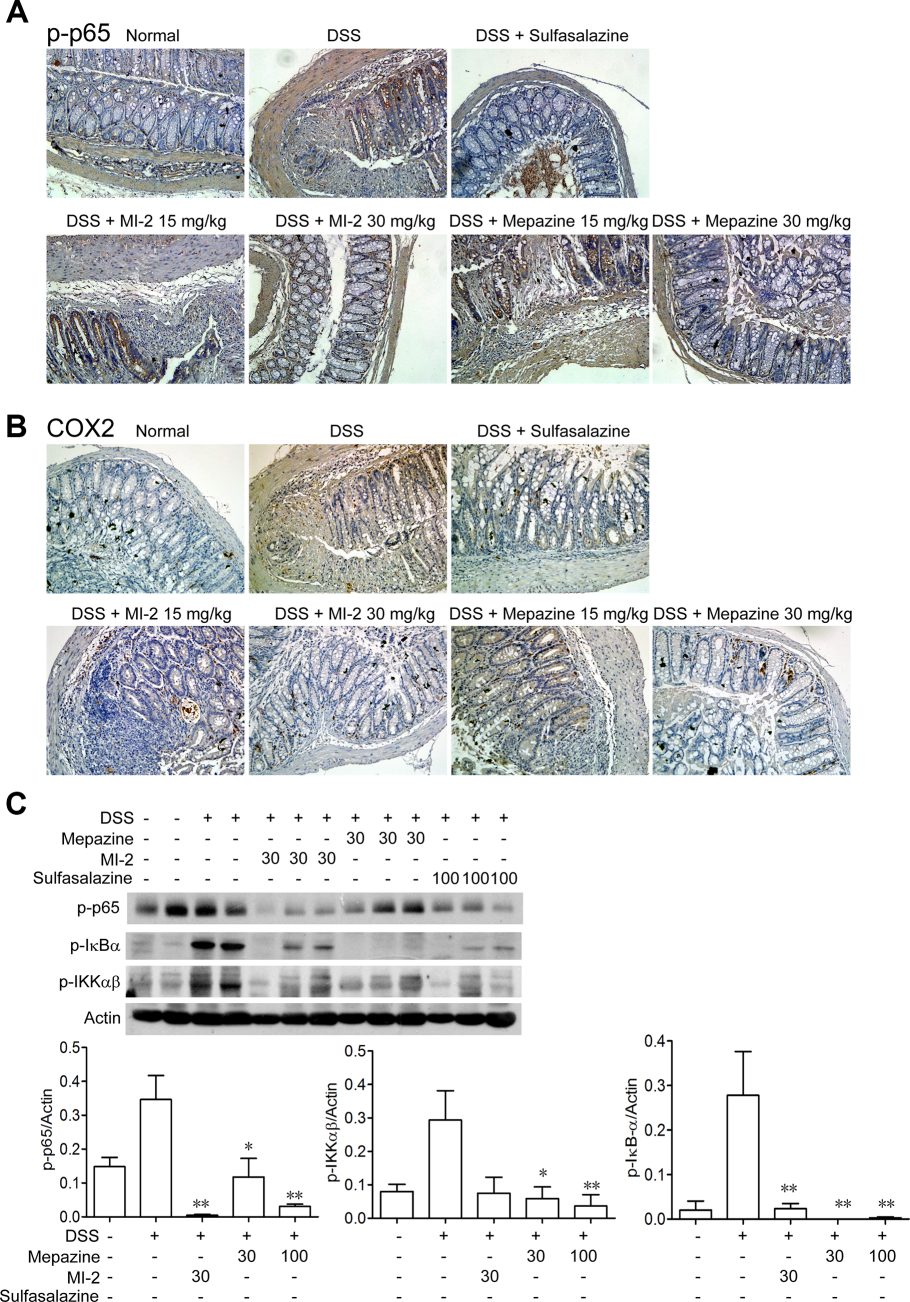
MALT1 inhibitors decreased activations of NF-κB signaling pathways in colon tissues from DSS-colitis mice (**A**, **B**) Serial sections of colon tissues were stained with p-p65 and COX2. (**C**) Colonic homogenate from each group of mice were subjected to western blot. Data are presented as means ± SEM. *P < 0.05, **P < 0.01 vs. DSS-treated group.

Activation of NF-κB play essential roles in transcriptional induction of various genes involved in inflammation, such as TNF, IL-1β and COX2 [30, 31]. As shown in Figure 4A and 4C, DSS treatment caused activation of NF-κB signaling evidenced by elevated IKKα/β, IKBα, p65 phosphorylation level in the colons from sick mice. MALT1 inhibitors treatment markedly reduced the activation or phosphorylation of these targeted proteins. Furthermore, COX2, an important mediator of the inflammatory which was the transcription product of p65, was also inhibited by mepazine and MI-2 (Figure 4B and 4C).

### MALT1 inhibitors inhibited the activation of NLRP3 inflammasome in DSS-induced colitis mice

**Figure 5:**
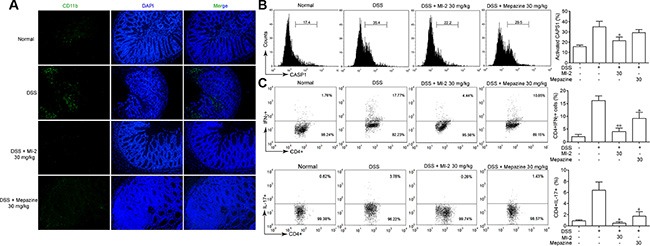
MALT1 inhibitors inhibited NLRP3 inflammasome activation in mice with DSS-induced colitis (**A**) Sections of colonic tissue were immunostained with DAPI (blue) and anti-CD11b-FITC (green) and observed by confocal laser-scanning microscope, 100×. (**B**) Spleen cells were extracted from colitis mice in each group at day 7 and CASP1 activation in CD11b+cells was analyzed by FACS staining. (**C**) Th1/Th17 differentiation in mice with DSS-induced colitis. Splenocytes was isolated from each group of mice and restimulated with PMA/ionomycin/monensin for 4 h. Th1 and Th17 cells were analyzed by intracellular staining of IFN-γ and IL-17A in the CD4 gate. Numbers in quadrants indicate percent cells in each throughout. Data are presented as means ± SEM. *P < 0.05 vs. DSS-treated group.

It has been documented that NLRP3 inflammasome may play crucial roles in DSS-induced colitis [11]. As shown in Figure 3, our data suggest that MALT1 inhibitors reduced colonic IL-1β/IL-18 production *in vivo*. To further investigate the mechanism of this reduced IL-1β/IL-18 production by MALT1 inhibitors, we examined macrophage infiltration in the colon tissues. Consistent with the local inflammation and other pathogenesis, an accumulation of large number of CD11b^+^ macrophages were observed in colonic samples from DSS mice (Figure 5A). In contrast, MALT1 inhibitors treatments led to significant reduction in macrophage infiltration, as evidenced by diminished FITC-positive cells by confocal immunofluorescent staining. In addition, CASP1 activation in mice spleen macrophages from MALT1 inhibitors-treated mice was also reduced (Figure 5B). Inflammasome-derived IL-1β are demonstrated to be necessary for the differentiation of T helper 17 (Th17) and T helper 1 (Th1) cells [32]. Consistently, MALT1 inhibitors also suppressed Th1 and Th17 differentiation in mice with DSS-induced colitis (Figure 5C). Our results showed that MALT1 inhibitors could suppress NLRP3 inflammasome activation in DSS-induced colitis model.

### MALT1 inhibitors inhibited the activation of NF-κB signaling *in vitro*

**Figure 6:**
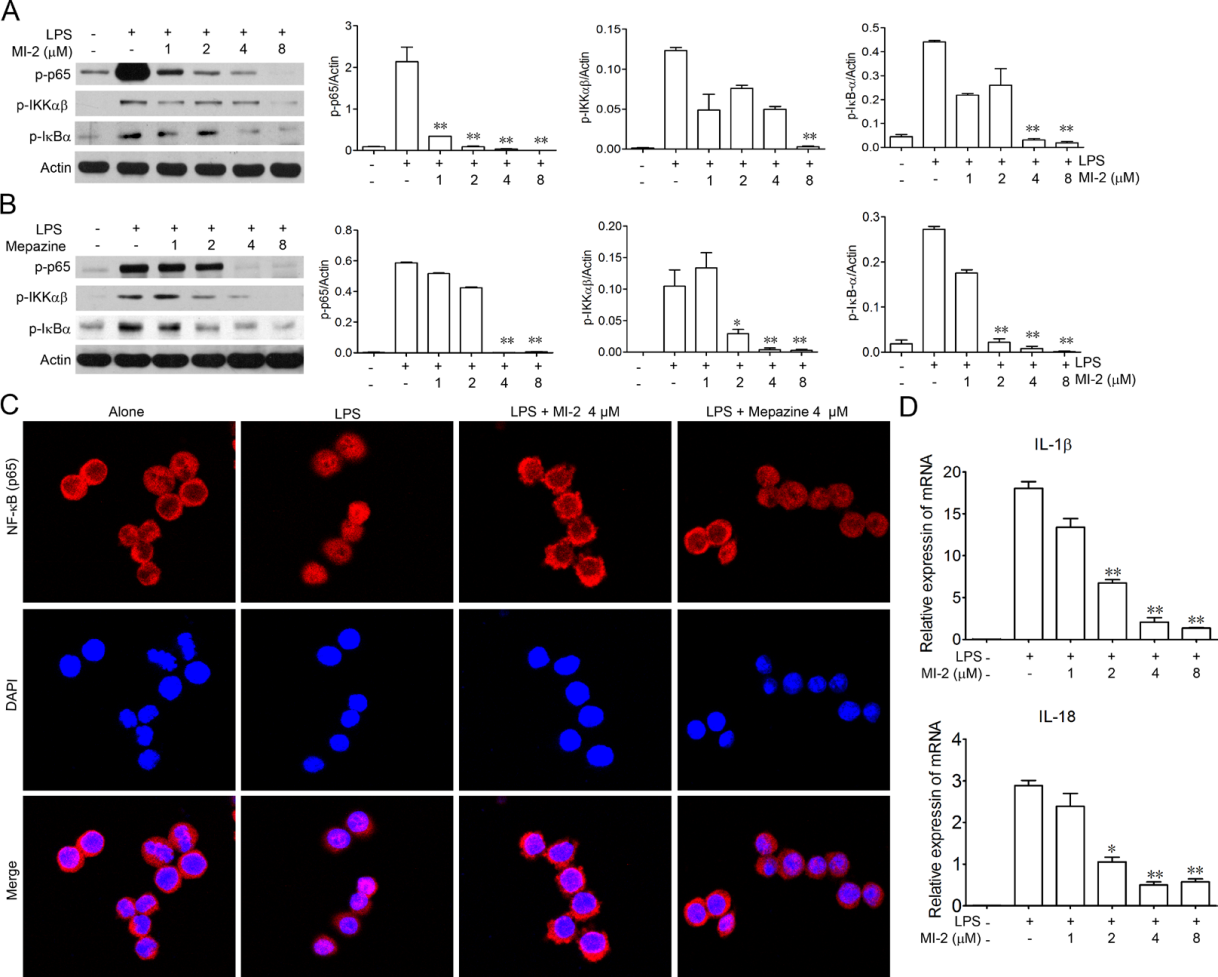
MALT1 inhibitors decreased activation of NF-kB signaling pathway *in vitro* (**A**, **B**) Peritoneal macrophage cells were treated with LPS alone or with MI-2, Mapezine for 1 h. Then proteins were collected and phosphorylation of p65, IκBα, IKKαβ were analyzed by western blot. (**C**) Peritoneal macrophage cells were treated with LPS alone or with MI-2 and Mapezine (4 μM) for 1 h, the localization of p65 was examined by immunofluorescence stain. (**D**) Peritoneal macrophage cells were treated with LPS alone or with MI-2 and Mapezine for 12 h, mRNA level of IL-1β and IL-18 were analyzed by RT-PCR. *P < 0.05, **P < 0.01 vs. LPS group.

To gain mechanistic insights on the anti-inflammation effects of MALT1 inhibition, we examined the effect of MALT1 inhibitors on the activation of NF-κB *in vitro*. As shown in Figure 6A and 6B, LPS-stimulated phosphorylation of IKKα/β was inhibited by MALT1 inhibitors in a dose-dependent manner, as well as the phosphorylation of IκBα. Consequently, the phosphorylation of the p65 was hampered by MALT1 inhibitors as well as the nuclear translocation of p65. As a result, mRNA level of IL-1β and IL-18 was diminished by MI-2 (Figure 6C).

### MALT1 inhibitors inhibited the activation of NLRP3 inflammasome *in vitro*

**Figure 7:**
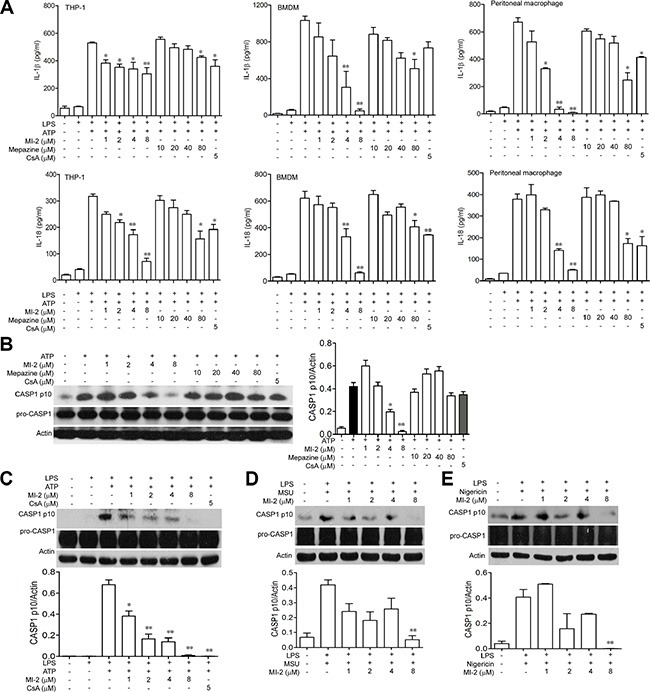
MI-2 inhibited IL-1β/IL-18 processing in THP-1 cells (**A**) LPS-primed THP-1/BMDM/peritoneal macrophage cells were treated with MI-2 or Mepazine (1, 2, 4, 8 μM) or CsA (5 μM) for 1 h, following by 1 h incubation of 5 mM ATP. Released IL-1β/IL-18 in the supernatant were analyzed by ELISA. Data are presented as means ± SEM of three different experiments. (**B**) LPS-primed THP-1 cells were treated with MI-2 or Mepazine (1, 2, 4, 8 μM) or CsA (5 μM) for 1 h, following by 1 h incubation of 5 mM ATP. Protein levels of pro-CASP1, cleaved CASP1, ASC and NRLP3 were determined by western blot. (**C**) LPS-primed THP-1 cells were treated with MI-2 or CsA (5 μM) for 1 h, following by 1 h incubation of 5 mM ATP. Protein levels of pro-CASP1, CASP1 p10, ASC and NRLP3 were determined by Western blot. (**D**, **E**) LPS-primed THP-1 cells were treated with MI-2 (1, 2, 4, 8 μM) or CsA (5 μM) for 1 h, following by 1 h incubation of 500 μg/ml MSU or 2 h incubation of 10 μM Nigericin. Protein levels of pro-CASP1, CASP1 p10, were determined by western blot. *P < 0.05, **P < 0.01 vs. LPS+ATP/MSU/Nigericin group.

IL-1β/IL-18 was processed as an inactive cytoplasmic precursor (pro-IL-1β/ pro-IL-18) which has to be cleaved by CASP1 to produce the mature active form. We examined the ability of MALT1 inhibitors to suppress the maturation of pro-IL-1β/ pro-IL-18 by NLRP3 inflammasome. As shown in Figure 7A, MI-2 exhibited a significant dose-dependent inhibition on IL-1β/IL-18 activation both in THP-1, BMDM and peritoneal macrophage while mepazine also slightly reduced IL-1β/IL-18 release at a high dose. We next examined the effect of MI-2 and mepazine on CASP1 activation. Our results showed that MI-2 inhibited the activation of CASP1 induced by addition of ATP *in vitro* but mepazine had no effect on CASP1 activation even at a high dose (Figure 7B). In addition, as shown in Figure 7C, 7D and 7E, MI-2 could inhibit the cleavage of CASP1 induced by various NLRP3 stimulators including ATP, MSU and Nigericin.

**Figure 8:**
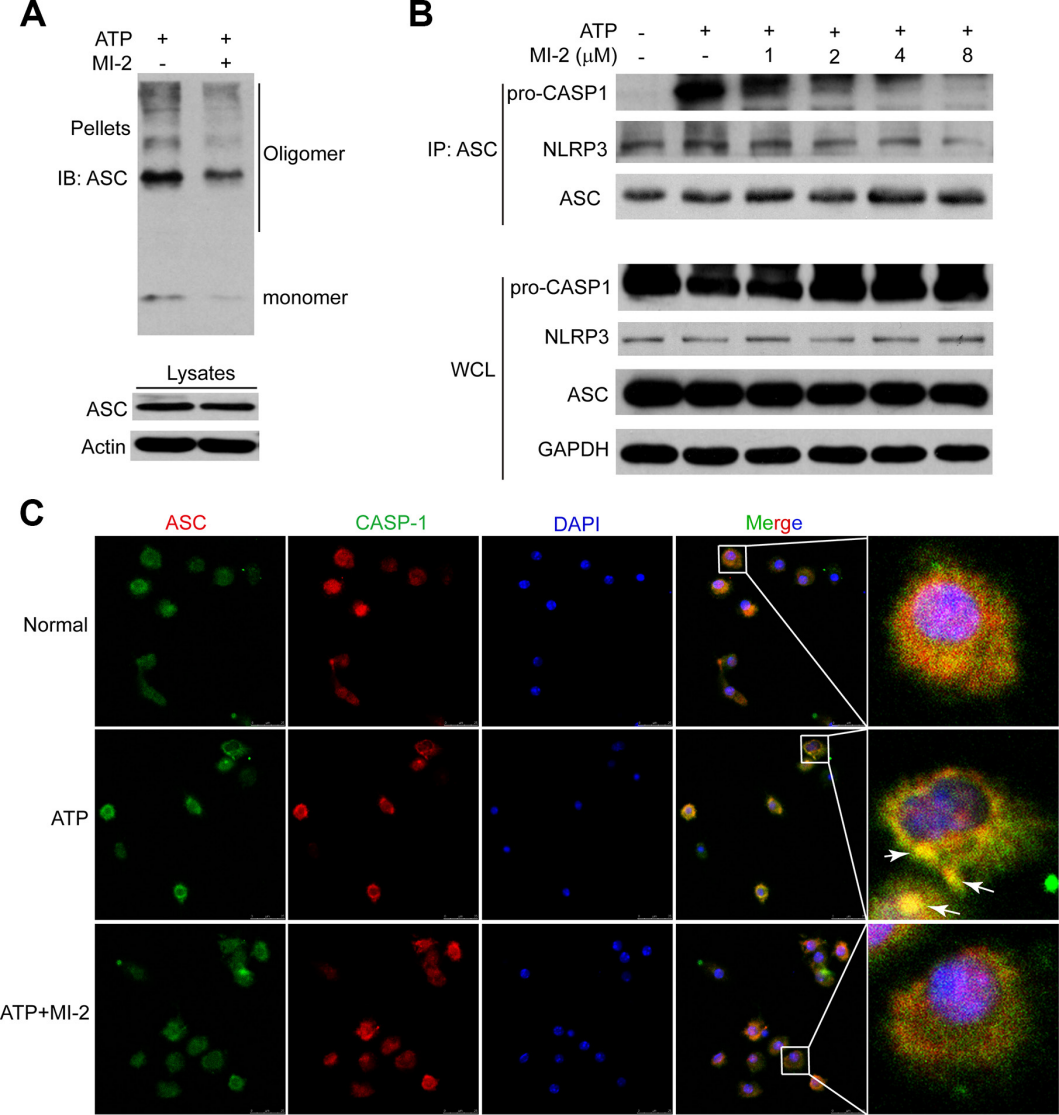
MI-2 suppressed NLRP3 inflammasome complex assembly induced by ATP (**A**) THP-1 cells were treated with MI-2 (4 μM) for 1 h, following by 1 h incubation of 5 mM ATP. ASC oligomerization and redistribution was determined by immunoblot of ASC in cross-linked pellets (upper panels) and in cell lysates (lower panels). (**B**) THP-1 cells were treated with MI-2, following by 15 min ATP treatment. Cell lysates were immunoprecipitated with anti-ASC. (**C**) LPS-primed adherent bone marrow derived macrophages (BMDM) were treated with MI-2 (4 μM) for 1 h, followed by 5 mM ATP treatment for 15 min. The treated cells were analyzed by immunofluorescence cytochemistry (×100).

Next, we sought to determine how MI-2 inhibited the activation of inflammasome *in vitro*. Upon ATP stimulation, NLRP3 recruits ASC and ASC recruits pro-CASP1, which results in autocatalysis and activation of CASP1, a key event in NLRP3 inflammasome activation. When cytosolic fractions from cell lysates were cross-linked, ASC oligomers were appeared after stimulation with ATP while MI-2 treatment reduced the formation of these oligomers (Figure 8A). Immunoprecipitation (IP) showed that MI-2 treatment inhibited the association of ASC with pro-caspase-1 CASP1 or NLRP3 (Figure 8B). Immunofluroscence analysis (Figure 8C) revealed that MI-2 markedly interrupted the co-localization of ASC and pro-CASP1, which subsequently inhibited cleavage of pro-CASP1. Collectively, these observations suggest that MI-2 can inhibit NLRP3 inflammasome-mediated CASP1 activation by decreasing the assembly of NLRP3/ASC/CASP1 complex.

## DISCUSSION

**Figure 9:**
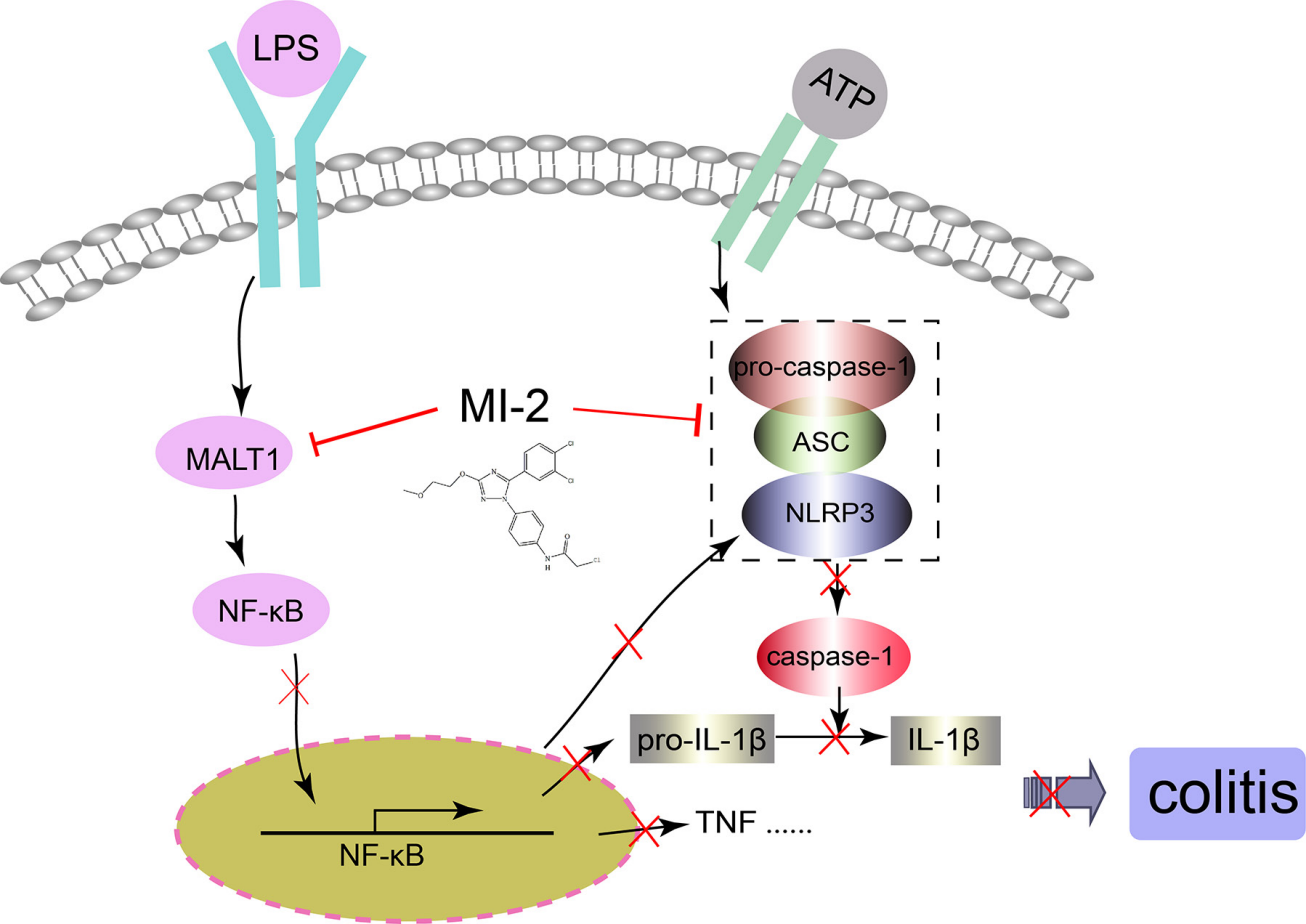
Illustration for the mechanism underlying MALT1 inhibitors for improvement of DSS-induced colitis in mice MALT1 inhibitor MI-2 can inhibit both NF-κB and NLRP3 inflammasome activation *in vivo* and *vitro*, thus decrease the protein and mRNA levels proinflammatory cytokines and finally alleviate colitis.

Nowadays, therapeutic options and approaches for inflammatory bowel disease continue to evolve. Generally, current treatment options for IBD are limited to reduction of symptoms and nonspecific immunomodulators such as salicylic acids, corticosteroids and TNF inhibitors. However, these currently available therapies are not sufficient due to either lack of efficacy or intolerable side effects and UC remains to be an area with huge unmet medical needs [33–35]. Our study here characterized that inhibitors of MALT1, would be safe and potent for the treatment of DSS-induced murine colitis by inhibiting NF-κB and NLRP3 inflammasome activation in macrophage (Figure 9). The conclusions were based on the following observations: first, intraperitoneal administration of MI-2 and mepazine at doses of 15–30 mg/kg significantly improved DSS-induced bloody stools, diarrhea and histopathologic indices in a dose-related manner. They inhibited the reduction of the colon length and the severity of the inflammatory lesions evaluated by a colon tissue histological assessment (Figure 1 and Figure 2). Second, the activation of NF-κB signaling pathway was significantly inhibited *in vivo* and *vitro* as proved by decreased phosphorylation of IKKα/β and NF-κB (p65) by MI-2 and mepazine treatment (Figure 4A and 4B) and reduced transcriptive productions of NF-κB including inflammatory cytokines (Figure 3A and Figure 4A). Third, the assembly of NLRP3/ASC/CASP1 complex activation of CASP1 and release of IL-1β/IL-18 were all suppressed by MI-2.

It is worth note that the production of proinflammatory cytokines is governed not only by TLR-NF-κB signaling but also by the protein family containing a nucleotide-binding domain and a leucine-rich repeat motif (NLR) [7, 36]. Together with ASC and CASP1, NLRP3 formed a complex named inflammasome to activate CASP1. In particular, IL-1β is translated by NF-κB as an inactive 31-kDa precursor (pro-IL-1β) after toll-like receptor stimulation (signaling I), and this precursor is cleaved to its activated 17-kDa form by the NLRP3 inflammasome-activated CASP1 (signaling II) [37]. Although there are controversies about the role of NLRP3 inflammasome during the process of colitis in various knockout mice [11, 38–40], many studies has proved that pharmacological blockage of NLRP3 activation and/or NF-kB can obviously ameliorate DSS colitis [41–45]. In the present study, our *in vitro* experimental results show that both compounds MI-2 and mepazine can inhibit the activation of NF-κB signaling thus reduced IL-1β/IL-18 secrtion (Figure 6) but inhibitory effect of MI-2 on NLRP3 activation is obviously better than that of mepazine. Using LPS plus ATP or MSU or Nigericin models of inflammasome activation, we investigated the influence of MI-2 on NLRP3 activation. MI-2 showed dose-dependent inhibition on the pro-CASP1 cleaved (Figure 7B) ASColigomerization and formation of NLRP3/ASC/pro-CASP1 complex (NLRP3 recruits ASC and ASC recruits pro-CASP1) is necessary for pro-CASP1 autocleavage. Immunoprecipitation and immunofluorescence assay manifested MI-2 treatment hampered ASC oligomerization and formation of NLRP3/ASC/pro-CASP1 complex (Figure 8). Compared these different effect on NLRP3 activation of MI-2 and mepazine, we deduced that MI-2 may have other targets on inhibiting NLRP3 inflammasome activation other than MALT1 or MI-2-triggered MALT1 may have some unknown functions related to NLRP3 inflammasome but yet we need more evidence.

Overall, our study found that new type inhibitor MI-2 has certain improvement effect on DSS-induced murine colitis by targeting MALT1-NF-κB and NLRP3 signal pathway, which suggests a new strategy for clinical treatment of colitis.

## MATERIALS AND METHODS

### Mice

Six- to eight-week-old female C57BL/6 mice were purchased from Model Animal Genetics Research Center of Nanjing University (Nanjing, China). Animal welfare and experimental procedures were carried out strictly in accordance with the Guide for the Care and Use of Laboratory Animals (National Institutes of Health, the United States) and the related ethical regulations of our university. All efforts were made to minimize animals' suffering and to reduce the number of animals used.

### Reagents

MI-2 and mepazine (chemical structure shown in Figure 1A, synthetic compounds provided by Eternity Bioscience Inc. NJ, USA) was dissolved at a concentration of 30 mM in 100% DMSO as a stock solution, stored at −20°C, and diluted with medium before each experiment. The final DMSO concentration did not exceed 0.1% throughout the study (all the control groups are composed of 0.1% DMSO). Cyclosporine A (CsA), phorbol myristate acetate (PMA), lipopolysaccharide (LPS) and adenosine triphosphate (ATP) were purchased from Sigma-Aldrich (St. Louis, MO). Dextran sulfate sodium (DSS, 36–50 kDa) was bought from MP Biomedicals (Aurora, OH). Myeloperoxidase (MPO) activity assay kit was purchased from Nanjing Jiancheng Bioengineering Institute (Nanjing, China). RPMI-1640, FBS, Alexa Fluor 546 Donkey Anti-Rabbit IgG and Alexa Fluor^®^ 488 Donkey Anti-Mouse IgG (H+L) were purchased from Life technology (Carlsbad, CA). Anti-phospho-IκBα, anti-phospho-IKKα/β, were purchased from Cell Signaling Technology (Beverly, MA). Anti-NLRP3, anti-phospho-p65 and anti-CASP1 were purchased from Epitomics (Burlingame, CA). Anti-ASC and anti-COX2 were purchased from Santa Cruz (Santa Cruz, CA). ELISA kits for murine TNF, IL-1β, IL-6, IFN-γ and human IL-1β were purchased from Dakewe Biotech Co. Ltd (Beijing, China). All other chemicals were purchased from Sigma-Aldrich (St. Louis, MO).

### Cell culture

Human THP-1 cells were purchased from Shanghai Institute of Cell Biology (Shanghai, China) and maintained in RPMI 1640 medium, supplemented with 100 U/ml of penicillin, 100 μg/ml of streptomycin and 10% fetal calf serum under a humidified 5% (v/v) CO_2_ atmosphere at 37°C. Bone marrow derived macrophages (BMDM) cells were isolated according to the following procedures. Bone marrow cells were isolated from C57/BL6 mice and cultured with DMEM supplemented with 10% fetal bovine serum and 20 ng/ml GM-CSF (Peprotech, Rock Hill, NJ). Culture fluid was exchanged to fresh culture medium every 3 days. Under these conditions, adherent macrophages were obtained within 7 to 8 days. Cells were harvested and seeded on 24-well plates. After culture for 6 h without GM-CSF, the cells were used for the experiments as bone marrow derived macrophages.

### Induction of colitis and treatment

Colitis was induced in C57BL/6 mice with 2.5% DSS (molecular weight 36–50 kDa) dissolved in drinking water (days 1–7). Normal mice were given water. Vehicle control (PBS), MI-2 (15, 30 mg/kg) and mepazine (15, 30 mg/kg) were given i.p from day 1 to day 10, respectively.

### Clinical scoring and histological analysis

Body weight, stool consistency and the presence of gross blood in feces and at the anus were observed everyday. The disease activity index (DAI) was calculated by assigning well-established and validated scores as reported previously [42]. At day 10 following induction of colitis, animals were sacrificed, the colon was removed and pieces of colonic tissue were used for *ex vivo* analysis. For histological analysis, part of the colon was fixed in 10% buffered formalin and embedded in paraffin. Sections were stained with H&E according to standard protocols. Histological evaluation of H&E-stained colonic sections was graded as follows: 0, no signs of inflammation; 1, low leukocyte infiltration; 2, moderate leukocyte infiltration; 3, high leukocyte infiltration, moderate fibrosis, high vascular density, thickening of the colon wall, moderate goblet cell loss, and focal loss of crypts; and 4, transmural infiltrations, massive loss of goblet cell, extensive fibrosis, and diffuse loss of crypts.

### Cytokine analysis by ELISA

Total protein was extract form colons of mice in each group by homogenating with lysis buffer. The homogenate was centrifuged at 12,000 *g* at 4°C for 15 min. The amount of total extracted protein was determined by BCA^™^ protein assay kit (Pierce, Rochford, IL). The amount of IFN-γ, IL-1β, IL-6, IL-17A, IL-18 and TNF in the colon homogenate was quantified by ELISA kit (Dakewe, Beijing, China).

### Intracellular staining

The intracellular expression of IL-17 and IFN-γ in CD4^+^ T cells was analyzed using Biolegend Intracellular staining kit according to the manufacturer's instructions. Lymphocytes obtained from the spleens were incubated with PMA (100 ng/ml)/ionomycin (1 μg/ml) and monesine (1 μg/ml) in complete media at 37°C for 4 h. Surface staining was performed with a CD4-FITC for 15 min at 4°C. After this, the cells were fixed and permeabilized with Fixation Buffer and Permeabilization Wash Buffer and intracellular cytokine staining was performed with IL-17A-PE and IFN-γ-APC for 20 min. Then the cells were analyzed by FACS.

### FACS staining for activated CASP1

Spleen cells were extracted from norma and drug-treated colitis mice at day 7 and stained with CD11b-PE and FLICA CASP1 (FITC) as according to the operation protocol (FLICA CASP1 assay Kit, Immunochemistry Technologies Company). Activation of CASP1-1 in CD11b^+^ cells was analyzed by FACS staining.

### Immunofluorescence histochemistry

CD11b^+^ macrophage infiltration analysis was performed on paraffin-embedded colonic tissue sections (5 μm). Briefly, the sections were deparaffinized, rehydrated and washed in 1% PBS-Tween. Then they were treated with 2% hydrogen peroxide, blocked with 3% goat serum and incubated for 2 h at room temperature with anti-CD11b FITC (1: 100). The slides were then counter-stained with DAPI for 2 min. The reaction was stopped by thorough washing in water for 20 min. Images were acquired by confocal laser-scanning microscope (Olympus, Lake Success, NY). Settings for image acquisition were identical for control and experimental tissues.

### Immunofluorescence cytochemistry

Bone marrow derived macrophages (BMDM) on coverslips were fixed in 4% paraformaldehyde (PFA), permeabilized with 0.5% Triton X-100 for 20 min and blocked with 3% BSA for 30 min. Cells were immunostained with monoclonal anti-ASC together with anti-CASP1 Ab overnight. Then Alexa Fluor 488-conjugated anti-mouse IgG and 594-conjugated anti-rabbit IgG (Life technology, CA) were immunostained for 2 h. The coverslips were counterstained with DAPI and imaged with a confocal laser scanning microscope (Olympus, Lake Success, NY).

### Western blotting

Cells were collected and lysed in the lysis buffer containing Triton X-100. After 10,000 g centrifugation for 10 min, the protein content of the supernatant was determined by a BCA^™^ protein assay Kit (Pierce, Rochford, IL). The protein lysates were separated by 10% SDS-PAGE and subsequently electrotransferred onto a polyvinylidene diuoride membrane (Millipore Corp., Bedford, MA). The membrane was blocked with 5% nonfat milk for 1 h at room temperature. The blocked membrane was incubated with the indicated antibodies. Protein bands were visualized using Western blotting detection system according to the manufacturer's instructions.

### Statistical analysis

Results were expressed as mean ± SEM of three independent experiments and each experiment included triplicate sets. Data were statistically evaluated by one-way ANOVA followed by Dunnett's test between control group and multiple dose groups. The level of significance was set at a *P* value of 0.05.
